# Modulation of the TGF-β signaling pathway by long noncoding RNA in hepatocellular carcinoma

**DOI:** 10.1186/s40364-020-00252-x

**Published:** 2020-12-01

**Authors:** Mengzhen Han, Zhibin Liao, Furong Liu, Xiaoping Chen, Bixiang Zhang

**Affiliations:** 1 Hepatic Surgery Center, Tongji Hospital, Tongji Medical College, Huazhong University of Science and Technology, 1095 Jiefang Avenue, Wuhan, 430030 China; 2 Hubei Key Laboratory of Hepato-Pancreato-Biliary Diseases, Wuhan, 430030 China

**Keywords:** Long noncoding RNA, TGF-β, Hepatocellular carcinoma, Therapy target

## Abstract

Hepatocellular carcinoma (HCC) is a type of liver cancer with poor prognosis. There have been demonstrated to exist many possible mechanisms in HCC tumorigenesis, and recent investigations have provided some promising therapy targets. However, further mechanisms remain to be researched to improve the therapeutic strategy and diagnosis of HCC. Transforming growth factor-β (TGF-β) is a pleiotropic cytokine which plays critical roles in networks of different cellular processes, and TGF-β signaling has been found to participate in tumor initiation and development of HCC in recent years. Moreover, among the molecules and signaling pathways, researchers paid more attention to lncRNAs (long non-coding RNAs), but the connection between lncRNAs and TGF-βremain poorly understood. In this review, we conclude the malignant procedure which lncRNAs and TGF-β involved in, and summarize the mechanisms of lncRNAs and TGF-βin HCC initiation and development. Furthermore, the interaction between lncRNA and TGF-β are paid more attention, and the potential therapy targets are mentioned.

## Introduction

Hepatocellular carcinoma (HCC), which accounts for 70–90% of primary liver cancer, is still the leading cause of cancer-related death worldwide, particularly in China [1, 2]. Most of HCC patients have suffered from cirrhosis caused by chronic hepatitis B and hepatitis C virus infection [3]. However, the molecular mechanisms in HCC pathogenesis remain not completely understood [4]. Early diagnosis is difficult for HCC, because there is always no obviously symptom in early stages of HCC. Although abundant therapeutic methods have been adopted, including surgical resection, chemotherapy, and targeted therapy, the prognosis of HCC is still poor [5]. In recent years, researches aimed at the roles of lncRNAs and TGF-β in HCC tumorigenesis. The results have also provided some possible therapy targets of HCC.

Transforming growth factor (TGF)-β is a multifunctional cytokine which has important effects on regulating cell growth, apoptosis and differentiation [6]. It belongs to the TGF-β superfamily, which consist of several related molecules, such as TGFβs (TGFβ1, TGFβ2 and TGFβ3), bone morphogenic proteins (BMPs), activins, growth and differentiation factors (GDFs) and anti-mullerian hormone (AMH) [7, 8]. Members of TGF-β superfamily pass signal through membrane-associated serine/threonine kinase receptors, and then activate SMAD transcription factors as well as other downstream molecules [9]. TGF-β proteins and their downstream molecules constitute the TGF-β signaling pathway, which attract many researchers to investigate its function.

Previous studies has indicated noncoding RNAs, containing microRNA (miRNA), circular RNA (circRNA), PIWI-interacting RNA (piRNA) and particularly long noncoding RNA (lncRNA), could participate in distinct physiological and pathological processes [10]. lncRNAs have been discovered to promote or inhibit some gene expression through diverse mechanisms [11]. In this review, we mainly concentrate on TGF-β signaling pathway, lncRNAs and their connection in HCC, which may provide some novel strategies for therapy and early diagnosis.

## TGF-β signaling pathway and its function in HCC

TGF-β signaling pathway can regulate a variety of cellular processes in human. TGFβs are important molecules in this signaling pathway. Before theirmaturation, TGFβs undergo several posttranslational modifications from precursor proteins of 390–412 amino acids. Firstly, two precursor proteins assemble to a dimer and subsequent cleaved by furin endopeptidase. After this process, latency-associated peptide (LAP) and the mature TGF-β protein come into being. Then, the LAP surround mature TGF-β and these two molecules form the small latent complex (SLC). Finally, SLC can bind to latent TGF-β binding protein (LTBP) via disulfide bonds, which is important in TGF-β proteins stability, assembly and secretion [12, 13].

After released into extracellular matrix, the TGF-β dimers bind to type II receptor and then type I receptor combines with type II receptor, which leads to the phosphorylation of type I receptors GS domain, SMAD2 and 3. Additionally, other branch of TGF-β superfamily like BMPs can bind to receptors and phosphorylate SMAD 1, 5 and 8 which are known as R-SMADs [14]. Furthermore, phosphorylated R-SMAD and SMAD4 form heteromeric complexes and then translocated into the nucleus [15]. Meanwhile, there are inhibitory SMAD proteins include SMAD6 and SMAD7 (I-SMADs), which are activated by TGF-β as a negative feedback system [16]. Taken together, the SMADs mentioned above make up the canonical TGF-β signaling pathway. However, there are some noncanonical downstream mediators which are induced by TGF-β independent on SMADs proteins, for example extracellular-signal regulated kinase (ERK), p38 mitogen-activated protein kinase (p38 MAPK), PI3k/AKT, RING domain-containing E3 ubiquitin ligases TRAF6, and several small GTPase such as Rho1, Rac and KRAS [17–21].

Disruption of TGF-β signaling pathway contributes to many diseases including all kinds of cancers [22]. In prior studies, abundance of results showed the TGF-β signaling pathway played pleiotropic roles in HCC, and exerts functions of either tumor suppressor or promoter [23]. In early stages HCC cells, activation of TGF-β lead to anti-proliferation response by inhibiting cell cycle at G1/S, or regulating p21 and p15 [24]. On the other hand, the overexpression of TGFβs in HCC suggests the malignant potential in complicated processes [25].

Firstly, TGF-β signaling participates in the process of cirrhosis and liver cancer initiation. Hepatic progenitor cells (HPCs) are stem cells in liver which can be activated under the circumstances of chronic liver injury. Recent studies indicated that HPCs could autocrine TGFβ1 and induce epithelial mesenchymal transition states through activation of SMAD2 and 3 in canonical signaling pathway. Furthermore, TGFβ1 secreted by HPCs can also activate autonomous secretion of connective tissue growth factor (CTGF) mediated by SMAD independent signaling, such as ERK, JNK and p38 MAPK signaling. Both of the mechanisms in HPCs activation are related to liver fibrosis and hepatocarcinogenesis [26, 27]. Likewise, known as stem cells of HCC, liver cancer stem cells (LCSCs) are also called tumor-initiating cells, which display abilities of self-renewal and differentiation and participate in new tumors generation [28]. In recent researches, it was reported that tumor-associated macrophages (TAMs) could secret TGFβ1 to promote LCSC properties by inducing EMT [29], and tumor-associated neutrophils (TANs) could secret TGFβ2 and BMP2, which induce miR-301b-3p and enhance stem cell characteristics in HCC [30]. Liver fibrosis is the most common precancerous change of HCC, and hepatic stellate cells (HSCs) have been identified as important myofibroblast progenitor cells in liver fibrosis. The TGF-β signaling pathway is involved in emergency of HSCs and the production of several extracellular matrix proteins. It was reported that bromodomain-containing protein 4 (BRD4) was critical in activation of HSCs, while experiments in murine models showed inhibitor of BRD4 could attenuate liver fibrosis and tumorigenesis through repressing TGF-β signaling pathway [31, 32]. These results indicated a tight connection between TGF-β and HCC initiation.

Secondly, TGF-β signaling also plays crucial roles in epithelial-mesenchymal transition (EMT). EMT is a process in which epithelial cells lose their adhesions between each other and require more mesenchymal characteristic. Meanwhile, it could be observed that some epithelial-typed proteins (E-cadherin, cytokeratin) are attenuated and some mesenchymal markers such as vimentin and N-cadherin are upregulated [33]. Therefore, EMT is closely associated with tumor metastasis [34, 35]. In previous studies, multiple findings have revealed that the process of EMT is regulated by the EMT-transcription factors (EMT-TFs) such as SNAIL, TWIST and ZEB1/2, which were reported as important inducers of HCC [36, 37]. The receptor tyrosine kinase Axl is upregulated in HCC cells. Axl can cause phosphorylation of SMAD3 linker region by binding to 14–3-3ζ, and result in upregulation of many downstream molecules of TGF-β signaling like Snail and MMP9 [38]. Platelet-derived growth factor (PDGF) and β-catenin are important hallmarks of EMT. Recent studies reported that TGF-β could induce autocrine and secretion of PDGF, then activate PI3K and Wnt/β-catenin signaling upon hepatocellular EMT [39]. Moreover, Protein Tyrosine Phosphatase Receptor Epsilon (PTPRe) which can bind to SMAD3 upon TGF-β stimulation, was observed to recruit SMAD3 to TGFBRI and induce EMT in HCC [40]. Overall, TGF-β signaling is the key inducer of EMT in HCC by abundant of pathways, including many different mechanisms of tumor progression.

Thirdly, since dysregulation of the immune system has been frequently reported in HCC, TGFβs are indicated as anti-inflammatory cytokines to inhibit anti-tumor immune responses [41]. TGF-β is critical for regulating immune cells differentiation, proliferation and development, including myeloid-derived suppressor cells (MDSCs), tumor-associated macrophages (TAMs), NK cells, and dendritic cells (DCs) [42]. Treg cells mainly play the inhibitory roles in HCC and lead to suppression of anti-tumor responses [43]. TGF-β can regulate Treg cells activity, and inhibit immune responses through suppressing effector T cells such as CD8+ cytotoxic T lymphocytes (CTLs), and restrain CD80/CD86 complex via CTLA-4 [42, 44, 45]. Likely, in NK cells, TGFβ1 can induce overexpression of CD96, and the reductions of CD226 and TIGIT, which break the balance and lead to dysfunction of NK cells in HCC [46]. Meanwhile, TGF-β signaling can also enhance PD-1 expression and suppress T-cells function [47]. Taken together, the previous experiments suggested the crucial function of TGF-β in immune inhibition.

**Fig. 1:**
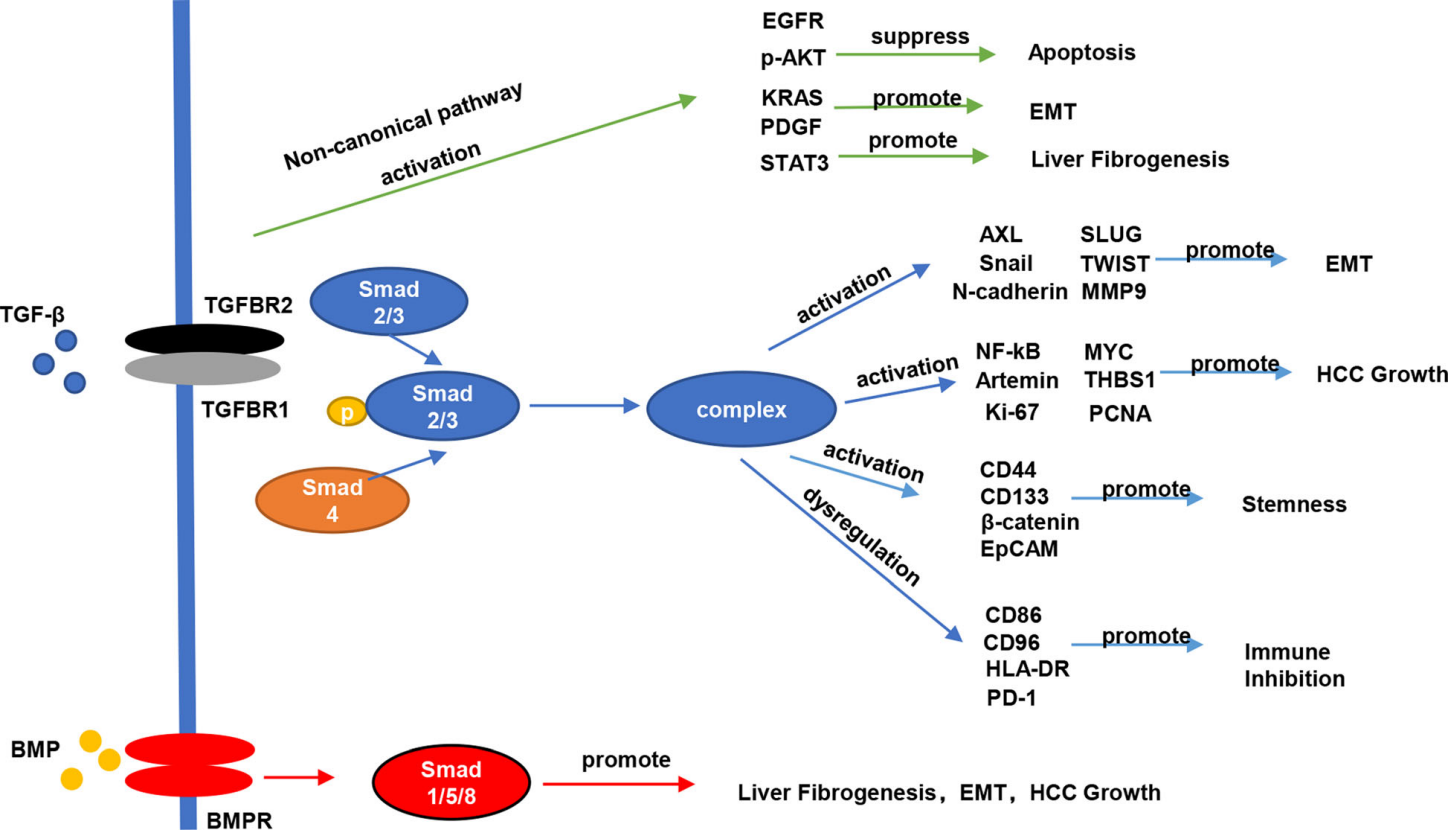
Outline of TGF-β signaling pathway regulating HCC initiation and progression. TGF-β affect downstream molecules through canonical (SMADs) and noncanonical (AKT, KRAS, EGFR, STAT3 and PDGF) pathway to regulate several HCC-related processes. Many downstream molecules of TGF-β signaling pathway were reported to involve in the processes including liver fibrogenesis (p38, STAT3),tumor growth (Ki-67, MYC, Artemin and NF-kB) stemness (CD44, CD133, EpCAM and β-catenin), EMT (N-cadherin, AXL and Snail), apoptosis (EGFR, p-AKT) and immune inhibition (CD86, CD96, PD-1 and HLA-DR)

The networks of TGF-β signaling pathway can be approximately divided into two groups. On the one hand, TGF-β proteins bind to TGBRs and phosphorylate either SMAD 2 and 3 or SMAD 1, 5, and 8, then they bind to SMAD4 and form a complex [14]. That is the canonical pathway. In HCC, dysregulation of TGF-β and SMAD proteins in all kinds of cells is able to cause tumor development and progression. For example, in recent studies, a kind of tumor-inducible, erythroblast-like cells (Ter-cells) were found gathering in the enlarged spleen, TGF-β and SMAD3 activation could induce Ter-cells generation, which was important in HCC progression [48]. Furthermore, TGF-β can stimulate β-catenin activation and translocate it into nucleus via SMAD2 and 3 in liver cancer stem cells [49]. On the other hand, noncanonical TGF-β signaling pathway, which is SMAD-independent also play crucial roles. In HCC cells, TGF-β signaling pathway mediates both pro- and anti-apoptosis process. The anti-apoptosis process is induced by epidermal growth factor receptor (EGFR), which is transactivated by TGF-β in the need of TACE/ADAM17 activation [50]. However, Daniel Caballero-Díaz et al. proved the importance of clathrin in induction of proliferative and anti-apoptosis signals by EGFR. In their findings, knockdown of clathrin could not affect phosphorylation of SMAD2, while the suppression of EGFR and AKT phosphorylation was observed. These results indicated that SMAD-independent pathway was involved in transactivation of EGFR [51]. Moreover, TGF-β signaling has been found to activate p38/JNK through TGF-β–activated kinase 1 (TAK1) and Mixed Lineage Kinase 3 (MLK3) in a non-SMAD pathway, holding the balance in cell fate [52, 53]. Likewise, TGF-β activates AKT signals by inducing autocrine regulation of PDGF, which lead to EMT process in HCC [39]. STAT3 activation also participate in stimulating HSCs upon TGF-β challenge, which leads to liver fibrosis [54]. In conclusion, TGF-β can modulate liver fibrosis and tumor initiation, EMT as well as immune inhibition through both SMAD and SMAD-independent pathways, which plays a dominant role in HCC formation. (Fig. 1).

## LncRNAs and the mechanisms of HCC tumorigenesis

Only 2% of the genome are able to encode for proteins [55], Non-coding RNAs which are longer than 200 bases are named as long noncoding RNAs (lncRNA) [56]. LncRNAs can be divided into five broad categories: (1) sense and (2) anti-sense which are transcribed from the sense or anti-sense strand of a protein encoding gene; (3) intergenic which locate between two genes; (4) intronic which locate in the whole intron and (5) bidirectional lncRNA when the expression of it and a neighboring coding transcript on the opposite strand locate within 1 kb from coding transcripts [57, 58]. The mechanisms based on the lncRNAs determine cells fates during their development, and dysregulation of lncRNA may result in many disorders [59]. In HCC, many researches also focus on lncRNAs to find out solutions for earlier diagnosis of liver cancer and effective treatment for advanced stages [60].

It was reported that these lncRNAs were involved in several malignant process of HCC. For example, some lncRNAs were demonstrated to be deregulated in LCSCs and HSCs and could cause the activation of HCC initiation and fibrosis, such as HULC and ANRIL [61, 62]. Likewise, lncRNAs can also participate in tumor growth and metastasis, through regulating EMT, cells proliferation and apoptosis [63–65]. Most of these lncRNAs could serve their functions by regulating the expression of target genes, including protein-coding genes or other non-coding RNAs [66]. Here, we have summarized four different mechanisms of lncRNAs according to their functions targeting DNAs, RNAs and proteins.

Firstly, lncRNAs can modulate gene expression through epigenetic modifications on promoters of target genes. For example, DLX6-AS1 is highly expressed in LCSCs and its overexpression contributes to methylation of cell adhesion molecule 1 (CADM1) promoter, which induce downregulation of CADM1 and activate STAT3 signaling pathway [67]. Likely, in HCC cells, there are similar mechanisms. BZRAP1-AS1 facilitates tumor angiogenesis via inducing methylation of the thrombospondin-1 (THBS1) promoter and repressing its transcription [68].Linc-GALH was observed to promote HCC metastasis through controlling the methylation status of Gankyrin, which was associated with ubiquitination of DNA methyltransferases 1 (DNMT1) [69]. Those results have demonstrated the function of lncRNAs in epigenetic regulation by interacting with DNAs.

Secondly, lncRNA can affect alterative splicing (AS) of pre-mRNA, which is important in regulation of gene function. Generally, AS is regulated by trans-acting protein factors [70]. Located in nucleus, some of trans-acting factors can combine with lncRNAs and lead to diversification of gene expression, which contribute to tumor growth and metastasis in colorectal cancer and breast cancer [71, 72]. Similarly, in HCC, MALAT1 has been found to bind to the splicing factor SRSF1, one of SR proteins, and upregulated the expression of it. Then SRSF1 could regulate AS of S6K1 to activate mTOR pathway, and enhance the production of antiapoptotic splicing isoforms as well [73], indicating that lncRNAs can modulate AS of pre-mRNA by binding to trans-acting proteins in HCC development.

Thirdly, some lncRNAs located in cytoplasm may exert their function as competitive endogenous RNA (ceRNA) in HCC development. In ceRNA network, lncRNAs sponge to specific microRNAs and competitively enhance the expression of target messenger RNAs. This mechanism is usually associated with tumor progression and chemotherapy resistance of HCC. For example, HULC was found to sponge to miR-2052 which could target the MET receptor tyrosine kinase. Consequently, overexpression of HULC could stimulate MET, which lead to tumor development and metastasis of HCC [74]. Likewise, EPB41L4A-AS2 was demonstrated to upregulate forkhead box L1 (FOXL1) via binding to miR-301a-5p [75]. The ceRNA network usually exists in malignant process of HCC.

Fourthly, lncRNAs can also bind to proteins, such as transcriptional factor, and modulate their activity and localization, which contribute to HCC progression. Linc00324 could interact with the transcription factor PU box binding protein (PU.1) and recruit it to promote expression of Fas ligand, which is significant to maintain the biological properties of LCSCs [76]. Conversely, downregulation of SLC2A1-AS1 in HCC could suppress the combination of signal transducer and activator of transcription 3 (STAT3), which leads to deregulation of FOXM1/GLUT1 axis and contribute to cells proliferation and metastasis [77]. Furthermore, Linc01093 could suppress HCC development by interacting with insulin-like growth factor 2 mRNA–binding protein 1, obstructing interaction between IGF2BP1 and glioma-associated oncogene homolog 1 (GLI1) mRNA [78]. These results showed the function of lncRNAs in HCC by binding to proteins. What’s more, some antisense lncRNAs can affect their corresponding mRNA to regulate malignant behavior of HCC. It was reported that LncHOXA10 could bind to SNF2L and recruited NURF chromatin remodeling complex to HOXA10 promoter, then activate HOXA10 in liver tumor initiating cells [79]. Likewise, PCNA-AS1 was observed to promote tumor growth by increasing PCNA mRNA stability [80]. In conclusion, lncRNAs show complicated mechanisms in tumorigenesis and might be possibly therapy targets of HCC (Table 1).

**Table 1:** LncRNAs and their mechanisms in HCC suppression and promotion

LncRNAs	Effect	Mechanism	Downstream Pathway
BZRAP1-AS1	Oncogene	Epigenetic modification	DNMT3b/THBS1
PVT1	Oncogene	Epigenetic modification	EZH2/MYC
Linc-GALH	Oncogene	Epigenetic modification	DNMT1/Gankyrin
DLX6-AS1	Tumor suppressor	Epigenetic modification	CADM1/Stat3
Linc00467	Oncogene	CeRNA	miR-509-3p/PDGFRA
Linc00346	Oncogene	CeRNA	miR-199a-3p/CDK1,CCNB1
HULC	Oncogene	CeRNA	miR-675/PKM2
		CeRNA	miR-2052/MET
RP11-422 N16.3	Tumor suppressor	CeRNA	miR-23b-3p/DMGDH
MALAT1	Oncogene	CeRNA	miR-140/VEGFA
		Alterative Splicing	SRSF1/S6K1
HAND2-AS1	Oncogene	Binding to proteins	INO80/BMPR1A
Linc00324	Oncogene	Binding to proteins	PU.1/FASL
Linc01093	Tumor suppressor	Binding to proteins	IGF2BP1/GLI1
SLC2A1-AS1	Tumor suppressor	Binding to proteins	STAT3/FOXM1/GLUT1
PCNA1-AS1	Oncogene	Antisense	PCNA1
LncHOXA10	Oncogene	Antisense	HOXA10

*BZRAP1-AS1* benzodiazapine receptor associated protein 1 antisense RNA 1; *DNMT3b* DNA methyltransferase 3B; *THBS1* thrombospondin-1; *PVT1* plasmacytoma variant translocation 1; *HULC* highly upregulated in liver cancer; Malat1, metastasis-associated lung adenocarcinoma transcript 1; *HAND2* heart and neural crest derivatives-expressed transcript 2; *DMGDH* Dimethylglycine dehydrogenase; *GLUT1* Glucose transporter type 1; *FOXM1* Forkhead box protein M1

## The interaction of TGF-β and lncRNAs in HCC: how they influence each other

Dysregulation of TGF-β and lncRNAs are important inducement in tumorigenesis. During recent decades, researchers have turn to pay more attention to the connection of these two elements, and these findings have revealed that lncRNAs and TGF-β could influence each other in several mechanisms of tumor initiation and progression. For example, SMAD2, 3 and 4 could transactivate HOTAIR by binding to its promoter site and promote metastasis of breast cancer [81]. X inactivate-specific transcript (XIST) could facilitate EMT induced by TGF-β through miR-367/141-ZEB2 axis in NSCLC [82]. Furthermore, studies of Pachera et al. showed lncRNA H19X, which was served as a critical regulator of TGF-β–driven tissue fibrosis, could be induced by TGF-β in a time- and dose-dependent manners in fibroblasts [83]. Similarly, in HCC TGF-β and lncRNAs could be able to influence each other through diverse mechanisms, which have been reported in HCC initiation and progression. In the investigation of Davide Degli Esposti et al., 5525 lncRNAs from different tissues were detected to associate with that in TGF-β and 57 of them differentially expressed in HCC compared with adjacent non-tumor tissues, These lncRNAs were co-expressed with genes about liver metabolism and cell cycle [84]. Generally, there are two ways to understand the relation between TGF-β and lncRNAs.

**Fig. 2:**
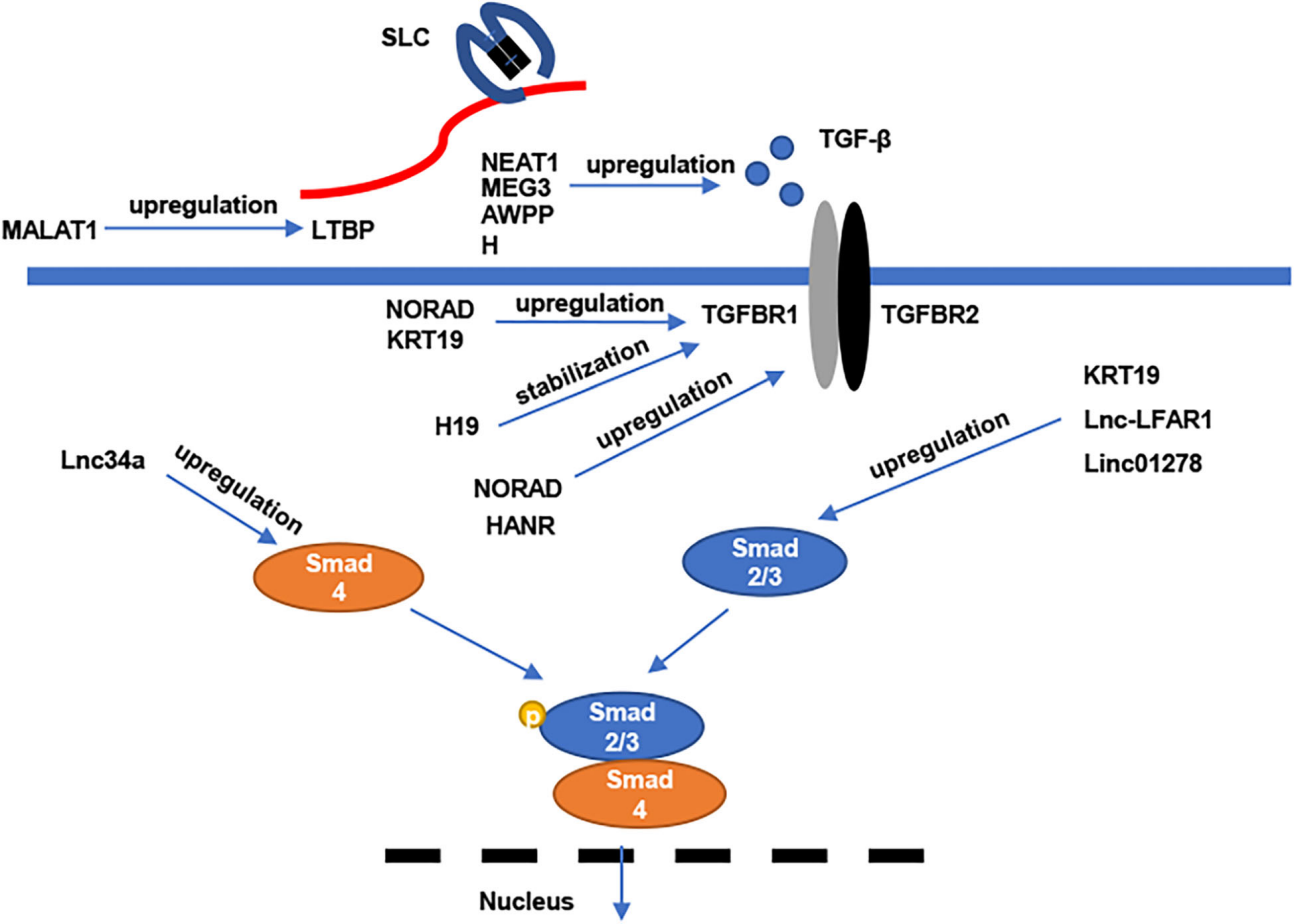
Function of lncRNAs in regulating TGF-β signaling pathway to affect HCC tumorigenesis. In HCC, regulation of many related or downstream molecules in TGF-β signaling pathway were reported to be relevant to specific lncRNAs: upregulation of LTBP (MALAT1), upregulation of TGFβ1 (NEAT1, MEG3 and AWPPH), upregulation of SMADs (Lnc34a, KRT19, Lnc-LFAR1 and LINC01278), upregulation or stabilization of TGFBRs (NORAD, KRT19, HANR and H19). These lncRNAs affect TGF-β signaling pathway by many action sites of downstream molecules

For one thing, lncRNAs can be served as upstream molecules of TGF-β signaling pathway by activating TGF-β and leading to malignant behaviors in HCC. Totally, some lncRNAs have been demonstrated to serve their function through targeting variety of molecules in TGFβ signaling pathway, including LTBP, TGFβs, TGFBR and SMAD proteins. As described earlier, these lncRNA affect TGF-β signaling pathway via similar mechanisms. Firstly, lncRNAs can modulate TGF-β/SMAD pathway through epigenetic modifications. For example, Lnc34a was recently indicated to interact with epigenetic regulators and methylate the miR-34a promoter to downregulate miR-34a expression, while miR-34a could target SMAD4 in TGF-β signaling pathway. Consequently, these findings revealed that Lnc34a could promote bone metastasis of HCC [85]. Secondly, lncRNAs can act as ceRNA to regulate TGF-β. NOARD is a lncRNA upregulated in HCC and correlated with the poor prognosis. In previous studies, NOARD was observed to competitively interact with miR-202-5p, which targets TGFBR1 and TGFBR2 [86]. HANR and SBF2-AS1 also exerted their functions through sponging to microRNAs which target TGFBRs [87, 88]. Meanwhile, Bai et al. showed that A1BG-AS1 could positively regulate SMAD7 and suppress HCC cells proliferation and invasion by sponging to miR-246a-5p [89]. Moreover, lncRNAs can directly interact with proteins to modulate TGF-β signaling pathway. For example, Lnc-LFAR1 could directly bind to SMAD2 and 3, and then enhance the expression of SMADs to promote their phosphorylation in liver fibrogenesis [90]. LncRNAs can also regulate BMP signaling through this mechanism. HAND2-AS1 was found to bind to INO80 and recruit the chromatin-remodeling complex to the promoter of BMPR1A and activate BMP signaling [91]. Dysregulation of these lncRNA can cause proliferation and metastasis of HCC cells and facilitate tumor initiation and progression (Fig. 2).

Furthermore, malignant functions of some lncRNAs can also be induced by TGF-β in HCC. LncATB is the most classical target lncRNAs of TGF-β signaling, named LncRNA activated by TGF-β. LncATB was firstly found to be upregulated in HCC and associated with poor prognosis [92, 93]. In HCC, LncATB could competitively bind to miR-200 family and consequently promote ZEB1 and ZEB2, which could lead to EMT and tumor metastasis [92, 94]. In another example, lncATB could also bind to autophagy-related protein 5 mRNA and promote its expression, in order to activate Hippo-YAP signaling, in order to promote autophagy [95]. Meanwhile, there are many other lncRNA targeted by TGF-β. UCA1 could be upregulated by TGFβ1 and promote tumor growth of HCC via influencing lactate production, glucose uptake and ATP production [96]. Liang et al. showed the overexpression of SLC7A11-AS1 by induction of TGF-β could facilitate HCC metastasis [97]. Moreover, LncRNA H19 expression was reduced by TGF-β through Sox2 and increased in TGFBR knocked out tumor-initiating hepatocytes (TICs). TUNEL assays showed that H19 knockdown caused apoptosis of TICs through TGF-β, and H19 is important in TICs progenitor capacity [98]. Taken together, these findings demonstrated that lncRNAs could be downstream molecules of TGF-β signaling to exert their functions in HCC initiation and progression.

Besides, there is a discovery of mechanism in LncRNA-hPVT1. In WANG et al. investigation, two independent lncRNAs microarray showed that LncRNA-hPVT1 could activate TGF-β signaling pathway. Simultaneously, LncRNA-hPVT1 could be induced by TGF-β. That means a feedback loop between these two elements in HCC progression [99] (Table 2).

**Table 2:** Downstream lncRNAs of TGF-β signaling in HCC

LncRNAs	Regulation by TGF-β	Downstream molecules	Function in HCC
LncATB	Upregulation	miR-200/ZEB1, ZEB2	EMT
		YAP, ATG5	Autophagy
HOTAIR	Upregulation	miR-145/P-gp, BCRP	Multidrug resistance
HCCL5	Upregulation	N-cadherin, E-cadherin	EMT
UCA1	Upregulation	HXK2	Tumor Growth
Linc-ROR	Upregulation	Caspase 8, GADD45B	Chemoresistance
PVT1	Upregulation	NOP2	Tumor Growth
H19	Downregulation	TP53/CDKI1A	Progenitor Capacity

*TGF-β* transforming growth factor-β; *ATG5* Autophagy Related 5; *HOTAIR* HOX transcript antisense RNA; *UCA1* urothelial carcinoma antigen 1; *HXK2* Hexokinase-2; *GADD45B* Growth arrest and DNA damage-inducible protein GADD45 beta; *PVT1* plasmacytoma variant translocation 1; *NOP2* Nucleolar Protein 2

## The possible therapeutic targets of HCC revealed in studies of TGFβ and lncRNAs

HCC is the most common type of primary liver cancer. The tumorigenesis of HCC is a complicated multi-step process containing multiple molecules and multiple signaling pathways. Therefore, investigation of molecule mechanisms in HCC initiation and progression might help us improve therapy strategies for HCC [100]. For example, recent studies revealed a novel molecule inhibitor of TGFBR1 named galunisertib. In phase2 clinical study of galunisertib in HCC patients and Child-Pugh A 5/6 or B7, the results showed that galunisertib could decrease level of AFP and prolong overall survival and progression-free survival [101]. And another TGFBR1 inhibitor LY2109761 has been observed to improve curative effect of TAE and suppressing metastasis [102]. In addition to the canonical pathway via TGFBR and SMAD proteins, noncanonical pathways such as p-AKT activated by TGF-β may be useful targets [103]. Moreover, as TGF-β signaling pathway suppress immune response, a dual-targeting inhibitor of TGF-β and PD-L1 was reported. It might enhance cytolytic ability of NK cells and reduce suppressive activity of Treg cells [104].

Combination treatment is now with the increasing appreciation in HCC because it has been shown to possess more sensitivity and less tolerance resistance than those in single drug. For example, an anti-malaria drug named artesunate was reported to collaborate with sorafenib and improve its efficacy in HCC through targeting ERK and STAT3, while combination with galunisertib could enhance the efficacy of sorafenib by delaying drug resistance of tumor [105, 106]. In our framework, we summarize the lncRNAs involved in TGF-β signaling pathway and provide possibility of collaboration between TGF-β inhibitors and lncRNAs or their downstream molecules in treatment of HCC. Firstly, several lncRNAs related to TGF-β have been found to influence chemotherapeutic resistance in HCC. For example, HOTAIR was reported to be upregulated by TGFβ1 and involved in TGFβ1-induced multidrug resistance (MDR) [107]. In Takahashi et al. studies, similar results happened on Linc-ROR, indicated that extracellular vesicles located Linc-ROR contribute to chemoresistance [108]. These lncRNAs could be directly targeted to enhance curative effect in combined pharmacotherapy. Secondly, some lncRNAs mentioned in TGF-β signaling pathway show relevant mechanisms with TGF-β proteins, which make it possible for synergistic anticancer effect in process of HCC tumorigenesis and progression. In another example, as an important mediate molecule in TGF-β signaling pathway, LncATB was demonstrated to competitively upregulate ZEB and trigger STAT3 signaling [92, 94]. Therefore, in our hypothesize, silencing of LncATB could suppress the ZEB and STAT3 signaling induced by TGF-β and might enhance the anticancer effect of TGF-β inhibitor. Moreover, it is worthy to notice that lncRNA H19 could be both upstream and downstream molecule of TGF-β [98, 109]. These findings might reveal multiple synergistic effect between TGF-β and H19. Thirdly, although there are also amounts of lncRNAs which are independent to TGF-β signaling pathway. Some of these lncRNAs are able to induce same downstream molecules with TGF-β, makes it possible for cooperation in HCC treatment. For instance, the TGFBR1 inhibitor galunisertib can block VEGF synthesis [110], while MALAT1 was observed to enhance VEGF production via inhibiting miR-140 and promoted angiogenesis of HUVECs in HCC [111]. These results showed possible combination between galunisertib and MALAT1 inhibitors in HCC treatment targeting angiogenesis. In addition, the TGF-β inhibitor SB431542 and CASC2c could repress ERK signaling and inhibit cell proliferation in HCC, which provide a novel therapeutic target for HCC [65, 112]. Taken together, by analyzing these findings, we hypothesize that many novel therapeutic strategies of combined pharmacotherapy for HCC could be found in collaboration between TGF-β and lncRNAs.

## Conclusion

Hepatocellular carcinoma is still a great menace for human-beings, and both therapy and early diagnosis methods need to be perfected. In our review, we paid considerable attention to lncRNAs and TGF-β signaling pathway, which have been studied in HCC for years. Totally, TGF-β has been proved to play distinct roles in HCC tumorigenesis through both canonical and noncanonical pathway. EMT is the major effect under induction of TGF-β signaling. Moreover, in tumor initiation and immune inhibition, TGF-β also serves as an important promoter. Other members of TGF-β superfamily like BMPs involve in HCC tumorigenesis as well.

When lncRNAs participate in dysregulation of TGF-β signaling pathway in HCC, they can exert their functions in varieties of different mechanisms. According to the subcellular location of these lncRNAs, they can affect DNA, RNA and proteins through epigenetic modification, alterative splicing, competitively sponge and directly binding. LncRNAs have been demonstrated to affect most of steps in TGF-β signaling pathway, such as TGF-β proteins, receptor and downstream SMAD2, 3 and 4. However, there are also many lncRNAs regulated by TGF-β. In this review, we have summarized several typical downstream lncRNAs of TGF-β signaling. These results are important complements of mechanisms in HCC initiation and progression.

In addition, investigations about dysregulation of TGF-β signaling pathway and lncRNAs may provide some promising therapeutic targets for HCC. Besides single drugs targeting TGF-β or some typical lncRNAs, we elucidate the possibility of combined pharmacotherapy by simultaneously targeting TGF-β signaling and special lncRNAs which could influence TGF-β signaling or be affected by the same downstream pathway of TGF-β. All in all, TGF-β and lncRNAs are promising therapeutic targets, and may provide some novel ideas about combined pharmacotherapy in HCC.

## Data Availability

Not applicable.

## Abbreviations

HCC: Hepatocellular carcinoma
TGF-β: Transforming growth factor-β
BMP: Bone morphogenic protein
LAP: Latency-associated peptide
SLC: Small latent complex
LTBP: Latent TGF-β binding protein
HPC: Hepatic progenitor cell
CTGF: Connective tissue growth factor
LCSC: Liver cancer stem cell
HSC: Hepatic stellate cell
TAM: Tumor-associated macrophage
TAN: Tumor-associated neutrophil
PDGF: Platelet-derived growth factor
EMT: Epithelial-mesenchymal transition
PD-1: Programmed cell death protein 1
EGFR: Epidermal growth factor receptor
STAT3: Signal transducer and activator of transcription 3
DNMT1: DNA methyltransferase 1
ERK: Extracellular regulated MAP kinase
AS: Alterative splicing
snRNP: Small nuclear ribonucleoprotein
MALAT1: Metastasis associated lung adenocarcinoma transcript 1
HULC: Hepatocellular carcinoma up-regulated long non-coding RNA
LncATB: Long noncoding RNA activated by TGF-β
